# Unmasking the Enigma of Weil's Disease: A Case Report

**DOI:** 10.7759/cureus.55124

**Published:** 2024-02-28

**Authors:** Yusuf Yalcin, Ibrahim Kamel, Harinder Singh

**Affiliations:** 1 Internal Medicine, Carney Hospital, Tufts University School of Medicine, Boston, USA; 2 Internal Medicine, Carney Hospital, Tufts Medical Center, Boston, USA; 3 Pulmonary and Critical Care Medicine, Carney Hospital, Tufts Medical Center, Boston, USA

**Keywords:** empiric therapy, diagnostic reasoning, clinical case report, severe respiratory failure, weil's disease, leptospirosis

## Abstract

We present the case of a 37-year-old male with Weil's disease, a severe form of leptospirosis, who presented without typical ecological risk factors. Initially manifesting as weakness, muscle aches, and fever, the patient rapidly deteriorated, necessitating ICU admission due to septic shock and respiratory failure. Despite initial diagnostic challenges, including normal initial imaging and inconclusive laboratory findings, a presumptive diagnosis of leptospirosis was made using Modified Faine's criteria. Empirical antibiotic treatment with doxycycline led to significant clinical improvement, highlighting the importance of early recognition and treatment in severe cases of leptospirosis. This case underscores the need for heightened clinical suspicion and the use of diagnostic scoring systems, even in atypical presentations, to facilitate timely intervention and improve patient outcomes.

## Introduction

Leptospirosis is a zoonosis caused by the pathological genus *Leptospira*. It is thought to be the most widespread zoonosis in the world but a rare disease in the United States (100 to 150 cases are reported annually) [1]. It is transmitted through direct or indirect contamination, mostly with rodent urine. Individuals who live in urban environments with low sanitation and poor housing are at high risk of rat exposure and leptospirosis [2]. The incubation period for leptospirosis is an average of seven to 14 days. The presentation of the disease can vary from a mild, influenza-like illness to multiorgan dysfunction. Weil’s disease is a severe form of this disease and is characterized by jaundice, hemorrhage, and acute renal failure. It develops in 5% to 10% of cases but is associated with a case fatality rate of 5% to 15% [3,4]. This case report aims to increase clinicians' awareness of the wide clinical spectrum of the disease and encourage the start of empirical antibiotic treatment for suspected patients.

## Case presentation

**Figure 1:**
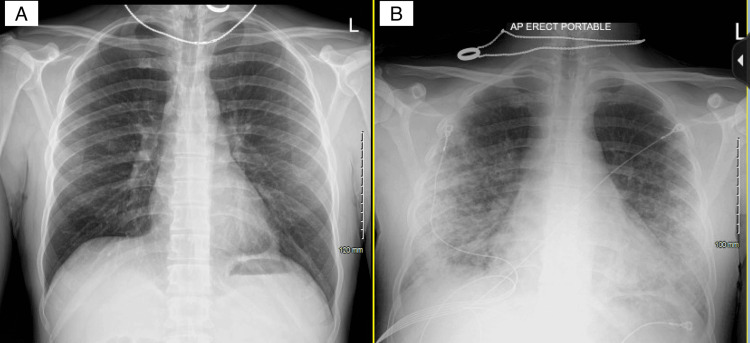
Comparison of the chest X-rays taken on the day of admission and day three of hospitalization A: The initial chest X-ray was unremarkable; B: Chest X-ray on day three of hospitalization shows bilateral airspace opacities

**Figure 2:**
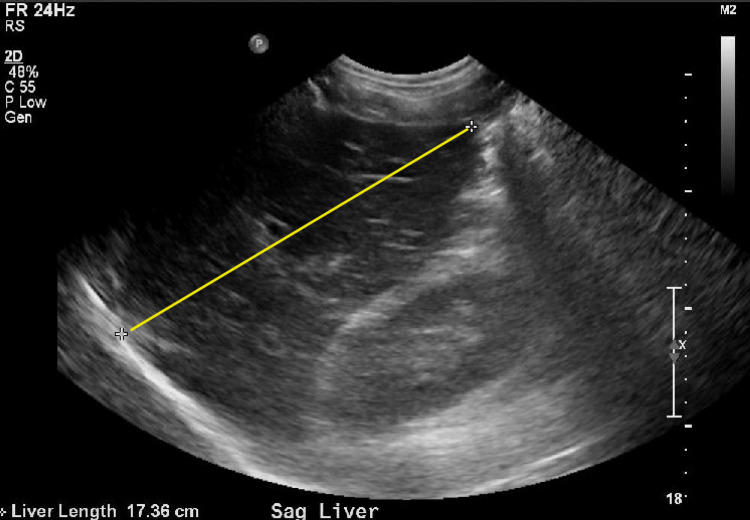
Right upper quadrant ultrasound of the abdomen shows a liver span of 17.36 cm

**Figure 3:**
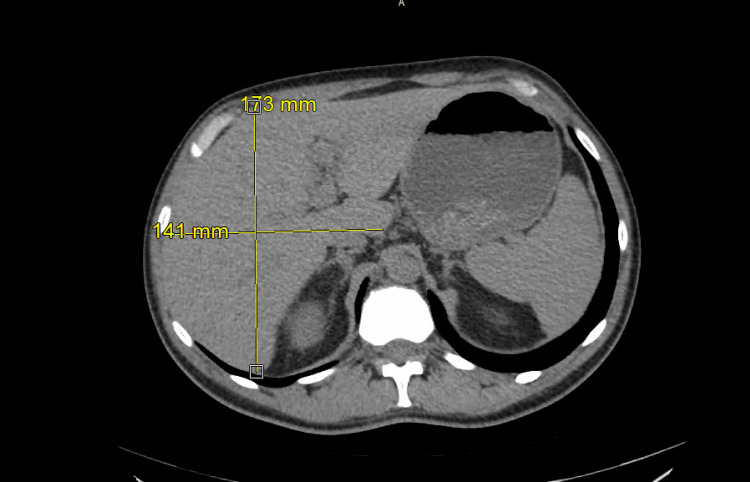
The CT abdomen shows hepatomegaly measuring 173 mm x 141 mm

A 37-year-old male with no known past medical history initially presented with weakness, muscle aches, and fever. Initial vital signs were stable except for sinus tachycardia. Physical examination was unremarkable except for scleral icterus. Pertinent positive lab results revealed leukocytosis, severe thrombocytopenia, elevated fibrinogen and D-dimer, acute kidney injury, and significantly elevated total and direct bilirubin. Pertinent negatives include relatively normal liver function tests, including the international normalized ratio (INR). Urine analysis was unremarkable. Acetaminophen level was negative. Initial imaging tests, including chest X-ray (Figure 1 A) and transthoracic echocardiogram (TTE), were normal. A CT of the chest, abdomen, and pelvis without contrast and right upper quadrant ultrasound (RUQUS) both showed mild hepatomegaly (Figures 2-3). On the second day, infectious diseases and gastroenterology were consulted, and a complete workup was done for common infectious etiologies such as influenza, COVID-19, respiratory syncytial virus (RSV), and rare etiologies, including rickettsia and leptospirosis. Our differential also included hemolysis, Wilson’s disease, and Fanconi’s syndrome. On the third day of admission, the patient was transferred to the ICU due to rapid deterioration, requiring high-flow oxygen and septic shock. The chest X-ray was remarkable for new bilateral airspace opacities (Figure 1 B).

**Figure 4:**
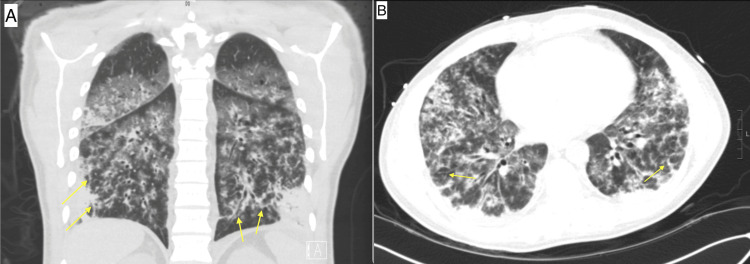
The CT scan of the chest shows multifocal consolidative and ground glass opacities with tree-in-bud nodularity (yellow arrows) in the coronal (A) and axial (B) views

The chest CT showed multifocal consolidative and ground-glass opacities with tree-in-bud nodularity (Figure 4). Due to severe shock, acute respiratory distress, impending respiratory failure, and the need for additional investigations, including liver biopsy as well as liver transplant evaluation if needed, the patient was transferred to a tertiary medical center ICU, where he was started on broad-spectrum antibiotics, vancomycin, and cefepime. A liver biopsy was obtained and showed signs of acute inflammation with lymphocytes and neutrophils. A presumptive diagnosis of leptospirosis was made using modified Faine's criteria, and the patient was started on doxycycline.

On the ninth day of admission, the *Leptospira* antibody IgM by enzyme-linked immunoassay (ELISA) returned positive, finally ending the mystery. Later, a sample was sent to the CDC, which confirmed the diagnosis by polymerase chain reaction (PCR). The patient responded well to antibiotic treatment, improving all parameters, including thrombocytopenia, liver function, respiratory failure, and kidney function.

## Discussion

Most leptospirosis cases are mild with flu-like symptoms, but in about 10% of cases also known as Weil's disease, mortality rates are higher. This is characterized by hepatic dysfunction associated with renal failure and hemorrhages [2]. Diagnosis is mainly based on clinical features and a history of risk exposure. The similarity of clinical symptoms with other infections such as dengue, influenza, malaria, and hepatitis adds another challenge to the mystery [5]. A scoring system using modified Faine's criteria was found to be a helpful tool for diagnosing leptospirosis in a resource-poor setting [6]. Although the scoring system exists to help guide the diagnosis, the rare state of this disease makes it a challenge to consider it the primary diagnosis. Diagnostic tests either show data of bacteria (culture, DNA, or spirochetes) in blood culture and PCR or dark-ground microscopy or antibodies against bacteria on microscopic agglutination tests and ELISA. However, the accuracy of these tests is variable. Currently, there is no vaccine for humans to prevent this infection [7].

What made this case incredibly complex was that the patient did not have any ecological risk factors. There was no exposure to epidemiological factors such as rainfall, contact with contaminated environments, or animal contact. High suspicion is essential to consider leptospirosis as a diagnostic hypothesis because, in its severe form, as in our patient, it can progress with fast clinical deterioration. Empirical treatment with antibiotics should be started when there is suspicion. More reliable prognostic tests and predictive models need to be explored, especially in patients without any risk factors, to avoid the disease's progression to its severe form.

## Conclusions

This case report highlights the intricacies of diagnosing and treating Weil's disease, a severe form of leptospirosis. Despite its rarity in certain regions, the potential for rapid clinical deterioration emphasizes clinicians' need to maintain a high index of suspicion. The modified Faine's criteria, introduced by the WHO, utilize various data points to assess the likelihood of leptospirosis, particularly in resource-poor areas. This scoring system incorporates clinical history, epidemiological factors, and laboratory data to make a presumptive diagnosis, with high scores indicating a probable case. Retrospectively applying these criteria to our patient revealed a high score, suggesting leptospirosis is likely. However, given the elusive nature of this disease, empirical antibiotic treatment should be promptly initiated upon clinical suspicion to prevent further morbidity and mortality. This report underscores the importance of heightened awareness, early recognition, and prompt intervention in effectively managing this condition.
